# An Inverse Relationship between Hyperuricemia and Mortality in Patients Undergoing Continuous Ambulatory Peritoneal Dialysis

**DOI:** 10.3390/jcm7110416

**Published:** 2018-11-05

**Authors:** Kuan-Ju Lai, Chew-Teng Kor, Yao-Peng Hsieh

**Affiliations:** 1 Department of Internal Medicine, Changhua Christian Hospital, Changhua 50006, Taiwan; 120752@cch.org.tw (K.-J.L.); 179297@cch.org.tw (C.-T.K.); 2 School of Medicine, Kaohsiung Medical University, Kaohsiung 80708, Taiwan; 3 School of Medicine, Chung Shan Medical University, Taichung 40201, Taiwan

**Keywords:** allopurinol, continuous ambulatory peritoneal dialysis (CAPD), cardiovascular disease, end stage renal disease, patient survival, uric acid

## Abstract

**Background:** The results have been inconsistent with regards to the impact of uric acid (UA) on clinical outcomes both in the general population and in patients with chronic kidney disease. The aim of this study was to study the influence of serum UA levels on mortality in patients undergoing continuous ambulatory peritoneal dialysis. **Methods:** Data on 492 patients from a single peritoneal dialysis unit were retrospectively analyzed. The mean age of the patients was 53.5 ± 15.3 years, with 52% being female (*n* = 255). The concomitant comorbidities at the start of continuous ambulatory peritoneal dialysis (CAPD) encompassed diabetes mellitus (*n* = 179, 34.6%), hypertension (*n* = 419, 85.2%), and cardiovascular disease (*n* = 186, 37.9%). The study cohort was divided into sex-specific tertiles according to baseline UA level. A Cox proportional hazard model was used to calculate hazard ratios (HRs) of all-cause, cardiovascular, and infection-associated mortality with adjustments for demographic and laboratory data, medications, and comorbidities. **Results:** Multivariate Cox regression analysis showed that, using UA tertile 1 as the reference, the adjusted HR of all-cause, cardiovascular, and infection-associated mortality for tertile 3 was 0.4 (95% confidence interval (CI) 0.24–0.68, *p* = 0.001), 0.4 (95% CI 0.2–0.81, *p* = 0.01), and 0.47 (95% CI 0.19–1.08, *p* = 0.1). In the fully adjusted model, the adjusted HRs of all-cause, cardiovascular, and infection-associated mortality for each 1-mg/dL increase in UA level were 0.84 (95% CI, 0.69–0.9, *p* = 0.07), 0.79 (95% CI, 0.61–1.01, *p* = 0.06), and 0.79 (95% CI, 0.48–1.21, *p* = 0.32) for men and 0.57 (95% CI, 0.44–0.73, *p* < 0.001), 0.6 (95% CI, 0.41–0.87, *p* = 0.006), and 0.41 (95% CI, 0.26–0.6, *p* < 0.001) for women, respectively. **Conclusions:** Higher UA levels are associated with lower risks of all-cause, cardiovascular and infection-associated mortality in women treated with continuous ambulatory peritoneal dialysis.

## 1. Introduction

Uric acid (UA) is the final product of a purine metabolism generated by xanthine oxidase or xanthine dehydrogenase in humans [1]. Rather than being ingested, uric acid is mainly produced by the liver from the metabolism of endogenous and exogenous purine products. Approximately two-thirds of the daily produced UA is excreted by the functioning of the kidneys, and the rest is largely excreted through the gastrointestinal tract. Elevated UA was significantly associated with rapid kidney function decline and incident chronic kidney disease (CKD) in the Jackson Heart Study of 3702 African Americans [2]. Therefore, hyperuricemia is frequently encountered in patients with renal failure, particularly those with advanced chronic kidney disease (CKD).

The burden on healthcare systems caused by chronic kidney disease (CKD) is increasing worldwide. Patients with CKD, including both those requiring hemodialysis and peritoneal dialysis, are at high-risk of cardiovascular (CV) events, and approximately 40% of patient deaths are due to cardiovascular disease (CVD) annually [3,4]. From the current literature, it has been equivocal regarding the role of UA in predicting the risk of cardiovascular disease and mortality. Some epidemiologic studies have identified an association between uric acid and CVD, as well as mortality in the general population and CKD patients [5,6,7,8,9]. Other studies have reported that this association is not independent and may be attributed to some concomitant cardiovascular risk factors, such as hyperglycemia, hypertension, and insulin resistance [10,11]. Given that UA is a potentially modifiable risk factor, clarifying its effects on clinical outcomes may change healthcare policies.

To date, only a handful of studies have investigated the association between UA and mortality in patients with end-stage renal disease (ESRD) treated with dialysis. Therefore, the purpose of the present study was to investigate the possible link between UA and mortality in patients undergoing continuous ambulatory peritoneal dialysis (CAPD) after adjusting for a variety of crucial variables. In addition, we also evaluated possible differences in sex with regards to the association between UA and mortality.

## 2. Patients and Methods

This study was carried out retrospectively at a single peritoneal dialysis (PD) center of Changhua Christian Hospital in Taiwan. From 1 January 2003 to 31 December 2014, patients who started CAPD as renal replacement therapy for ESRD were screened for this study. The enrollment criteria included incident patients of at least 18 years of age who had survived on CAPD for at least 3 months. Eligible patients were followed up from the date of commencing CAPD and the end of the study was on 31 December 2015 when the clinical outcomes were censored. CVD and infection were the two leading causes of death in our patients. Therefore, the primary outcome of interest was all-cause mortality, and the second outcomes of interest were CV and infection-associated mortality. CV mortality was defined if a death was attributed to any of the following cardiovascular events: coronary events, cardiomyopathy, cardiac dysrhythmia, sudden cardiac death, congestive heart failure, ischemic brain injury, cerebrovascular accident and peripheral vascular disease. Infection was the cause of death if a patient directly died from sepsis. The cause of death was determined by reviewing the discharge diagnosis and hospitalization summary in electronic medical records. The study protocol was approved by the institutional review board of our hospital, and the study was conducted in compliance with the declaration of Helsinki.

Baseline characteristics of the whole cohort were obtained from the hospital’s electronic database. Demographic and clinical data were recorded at the initiation of PD, and included age, sex, status before PD (pre-dialysis CKD, hemodialysis, and failed renal transplantation), body mass index (BMI), medications, comorbidities, and smoking status. The comorbidities included diabetes mellitus (DM), hypertension, CVD and Charlson Comorbidity Index, which is one of the most commonly used scoring systems to predict the risk of mortality. Blood tests, consisting of white blood cell (WBC) counts, hemoglobin, total cholesterol, triglyceride, calcium, phosphate, alkaline phosphate (ALP), uric acid, glutamic-pyruvic transaminase (GPT), intact parathyroid hormone (iPTH), ferritin, transferrin saturation, serum albumin, blood urea nitrogen (BUN), and creatinine were checked at the laboratory of Changhua Christian Hospital. The use of medications was recorded based on prescriptions, and included angiotensin-converting enzyme (ACE) inhibitor, angiotensin II receptor blocker (ARB), hypouricemic agent (allopurinol), erythropoiesis stimulating agents (ESA), loop diuretic (furosemide), calcium channel blocker (CCB), vitamin D, and statins. Information on PD-related parameters included daily ultrafiltration, total Kt/V urea, residual renal function (RRF), daily urine volume, normalized protein nitrogen appearance (nPNA) and dialysate-to-plasma creatinine ratio (D/P creatinine) at 4 h. RRF, in milliliters per minute per 1.73 m^2^, was derived from averaging the values of creatinine and urea clearance and standardized for body surface area.

## 3. Statistical Analysis

The patients were classified into sex-specific tertiles according to the serum UA level: for men, tertile 1 ≤ 6.8 mg/dL; tertile 2 = 6.9–8 mg/dL; tertile 3 ≥ 8.1 mg/dL; and for women, tertile 1 ≤ 6.5 mg/dL; tertile 2 = 6.6–7.6 mg/dL; tertile 3 ≥ 7.7 mg/dL. Summary statistics are presented as mean ± standard deviation (SD) for continuous variables of normal distribution, median with interquartile range (IQR) for continuous variables of skewed distribution, or as number (*n*) and percentage (%) for categorical variables, unless otherwise indicated. Differences in categorical variables among the three groups were tested using the chi-square test or Fisher’s exact test. Continuous data with normal distribution were compared by analysis of variance (ANOVA), and skewed continuous data by the Kruskal-Wallis test.

Multivariate linear regression analysis with backward elimination procedure and removal criterion for probability of F-distribution ≥0.100 was used to evaluate associations between clinical parameters at baseline and UA, and to estimate the expected change in serum UA level in relation to changes in significant clinical parameters. Associations between UA and the outcomes of interest were evaluated using Cox proportional hazard models, which were constructed using five levels of adjustments: model 1, adjusted for sex, age, the status before PD, smoking status, and BMI; model 2, adjusted for model 1 covariates plus medications; model 3, adjusted for model 2 covariates plus comorbidities; model 4, adjusted for model 3 covariates plus PD-related parameters; model 5, adjusted for model 4 covariates plus laboratory data. The results are shown as hazard ratios (HRs) and 95% confidence intervals (CIs).

To strengthen our findings, we conducted three sensitivity tests. First, Cox regression analysis was repeated with UA as a continuous variable with HRs presented per 1-mg/dL increase in serum UA level by sex. Second, all of the patients were dichotomized into either hyperuricemia or normouricemia groups by sex to examine the relationship between hyperuricemia and mortality. Hyperuricemia was defined as a UA level >7 mg/dL for men and >6 mg/dL for women. Third, the relationship between UA and mortality was calculated after excluding the patients taking allopurinol at entry to the study to mitigate the confounding effect of hypouricemic agents on serum UA level. In addition, we also performed stratified analyses by nutritional markers, including BMI, cholesterol, albumin, nPNA, phosphorus, and creatinine. Statistical significance was set at a *p* value < 0.05 for the analysis. All statistical analyses were conducted using IBM SPSS Statistics for Windows, Version 22.0 (IBM Corp., Armonk, NY, USA).

## 4. Results

### 4.1. Patient Characteristics

Four hundred and ninety-two CAPD patients were enrolled in this study, with a median follow-up period of 36.4 months. Most (81.9%) of our study participants commenced CAPD as the first renal replacement therapy, i.e., pre-dialysis status. The baseline patient characteristics according to the sex-specific tertiles of UA are shown in Table 1. The mean age of the patients was 53.5 ± 15.3 years, with 52% being female (*n* = 255). The concomitant comorbidities at the start of CAPD encompassed diabetes mellitus (*n* = 179, 34.6%), hypertension (*n* = 419, 85.2%), and cardiovascular disease (*n* = 186, 37.9%). The median UA level was 7.2 (IQR, 6.3–8.2) mg/dL for all patients, and the men had a significantly higher UA level than the women (7.4 (6.6–8.3) mg/dL vs. 7 (6.1–8) mg/dL, respectively; *p* = 0.004). A total of 126 participants (25.6%) were taking hypouricemic agent (allopurinol) at baseline. One hundred and twenty-seven patients died during the study period, including 74 from cardiovascular disease and 41 from infection.

### 4.2. Uric Acid (Sex-Specific Tertiles) and All-Cause Mortality

**Figure 1:**
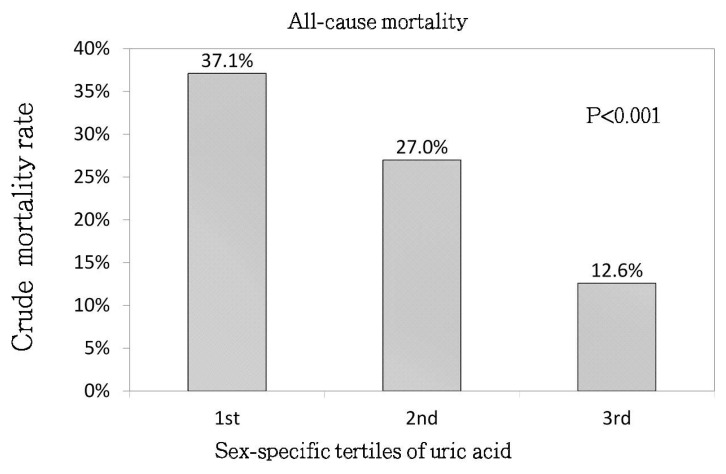
Crude analysis of all-cause mortality rate between the sex-specific tertiles of serum uric acid levels (*p* < 0.001).

As shown in Figure 1, the all-cause mortality rate showed a stepwise decrease from UA tertile 1 to UA tertile 3 (37.1%, 27%, 12.6%, respectively; *p* < 0.001). The associations between UA tertile and all-cause mortality by Cox regression analysis are shown in Table 2. Compared with the UA tertile 1, UA tertile 3 was associated with a lower risk of overall mortality in the fully adjusted model (model 5; adjusted HR (aHR) = 0.4, 95% CI, 0.24–0.68, *p* = 0.001).

### 4.3. Uric Acid (Sex-Specific Tertiles) and CV Mortality

**Figure 2:**
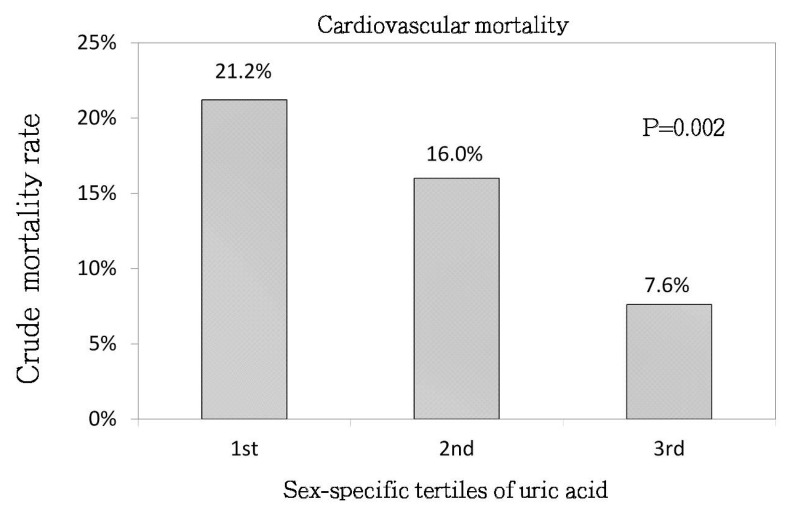
Crude analysis of cardiovascular mortality rate between the sex-specific tertiles of serum uric acid levels (*p* = 0.002).

As shown in Figure 2, the CV mortality rate showed a stepwise decrease from UA tertile 1 to UA tertile 3 (21.2%, 16%, 7.6%, respectively; *p* = 0.002). The associations between UA tertiles and CV mortality are shown in Table 2. Using UA tertile 1 as the reference, the aHR for UA tertile 3 was 0.4 (95% CI, 0.2–0.81, *p* = 0.01) with respect to CV mortality.

### 4.4. Uric Acid (Sex-Specific Tertiles) and Infection-Associated Mortality

**Figure 3:**
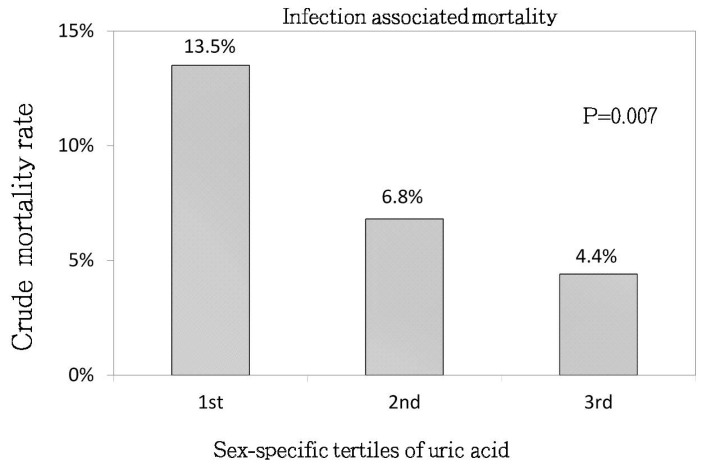
Crude analysis of infection-associated mortality rate between the sex-specific tertiles of serum uric acid levels (*p* = 0.007).

As shown in Figure 3, the infection-associated mortality rate showed a stepwise decrease from UA tertile 1 to UA tertile 3 (13.5%, 6.8%, 4.4%, respectively; *p* = 0.007). The associations between UA tertiles and infection-associated mortality are shown in Table 2. There were no statistically significant differences in the risk of infection-associated mortality among the UA tertiles.

### 4.5. The Association between Mortality and Non-Sex-Specific Uric Acid Tertiles

We also evaluated the relationship between mortality and UA by non-sex-specific tertiles. As shown in Table 3 higher UA levels were associated with lower risks of all-cause, CV, and infection-associated mortality.

### 4.6. Sensitivity Analysis

A similar association between UA and mortality was noted when we treated UA as a continuous variable (Table 4). In the fully adjusted model, the aHRs of all-cause, CV, and infection associated mortality for each 1-mg/dL higher UA level were 0.84 (95% CI, 0.69–0.9, *p* = 0.07), 0.79 (95% CI, 0.61–1.01, *p* = 0.06), and 0.79 (95% CI, 0.48–1.21, *p* = 0.32) for men and 0.57 (95% CI, 0.44–0.73, *p* < 0.001), 0.6 (95% CI, 0.41–0.87, *p* = 0.006), and 0.41 (95% CI, 0.26–0.6, *p* < 0.001) for women, respectively. After excluding 126 patients who received allopurinol treatment, the significant association remained in the women but not in the men (Table 3). Based on the pre-defined sex-specific UA levels to classify the patients as being hyperuricemic or non-hyperuricemic, hyperuricemia was clearly correlated with a lower risk of overall mortality, CV mortality, and infection-associated mortality in the women, but not in the men.

### 4.7. The Correlation of UA with Other Parameters

Table 5 listed the significant association between UA and clinical parameters at baseline by multivariate linear regression analysis. Serum UA level was positively correlated with male gender, albumin, BUN, iPTH, phosphorus, triglyceride, CV disease, and residual renal function, and negatively correlated with age and 24-h urine output.

### 4.8. Stratified Analyses

Table 6 showed the results of the stratified analyses by BMI, cholesterol, albumin, nPNA, phosphorus, and creatinine. The median values of those parameters were used to divide the entire cohort into two subgroups. Nearly all the *p* values for interaction showed non-significant results, indicating no significant interaction effects between UA and these nutritional markers.

## 5. Discussion

In this study, we examined the associations between UA and all-cause, CV, and infection-associated mortality risk at a single PD center in Taiwan. The major finding of this study is that a higher UA level (UA tertile 3) was associated with a lower risk of all-cause, CV, and infection-associated mortality only in the female ESRD patients treated with CAPD, independent of several confounding factors. This observation was further verified by similar results noted when UA was treated as a dichotomous or continuous variable. Furthermore, the association remained significant after excluding the patients taking hypouricemic agents. These findings are in contrast to previously published studies on PD patients, and imply that hyperuricemia per se may protect against the risk of mortality in PD patients.

To date, only few investigations have evaluated the impact of UA on mortality in PD patients. One study, conducted in Sweden, comprised 294 subjects starting renal replacement therapy, 161 of whom were started on peritoneal dialysis and the rest were started on hemodialysis [12]. All the patients were evaluated as a whole. During a mean follow-up of 27 months, most of the patients changed their initial dialysis modality with the majority being on hemodialysis at the end of study. They concluded that UA showed a J-shaped relationship with all-cause mortality, with the greatest risk in the 5th quintile. Another retrospective study involved 156 PD subjects conducted in China, which excluded a PD duration of less than 3 months, a history of kidney transplantation and cirrhosis [13]. UA levels were checked both before starting peritoneal dialysis and 3 months following PD for the analysis with mortality. They also demonstrated a J-shaped UA-mortality association that a higher serum UA level is an independent risk factor for all-cause mortality in PD patients. Later, Xia et al. conducted a large investigation of 985 PD patients in South China and found that an elevated level of UA was an independent risk factor for all-cause and CV mortality in men, but not in women, treated with PD [14]. Therefore, the association between UA and mortality may largely be influenced by gender. However, a multicenter study of 2264 patients on chronic PD in China reported contrasting results, in that the prognostic value of UA for all-cause and CV mortality weakened or disappeared after adjusting for uremia-related and traditional CV risk factors [15]. More recently, UA was reported to be a predictor for cardiovascular mortality in male PD patients with diabetes [16].

The reasons for the differences in the impact of UA on patient survival between male and female patients have yet to be clarified, and may be due to patient ethnicity, differences in the follow-up period, differences in the adjusted confounders, and differences in serum UA levels between men and women. A study reported a significant relationship between UA and CV mortality both in women and men after a longer follow-up period, while the previous study of the same cohort only reported the association merely in women [17]. Sex hormones may also result in different results between men and women. Estradiol, progesterone, and testosterone have been reported to affect UA transporters in mice kidneys, and serum UA levels have been reported to be higher in post-menopausal than pre-menopausal women [18,19]. Furthermore, the differences in sex may also indicate that women are more susceptible to UA-associated damage than men, as evidenced by studies on arterial stiffness, silent brain infarction and internal carotid artery resistive index [11,20,21,22]. However, the exact reasons for this relationship remain to be clarified.

The contradictory results relating UA to outcomes were partly attributed to the dual effects of UA on CV outcomes. Several mechanisms were proposed to explain the detrimental effects of UA. First, hyperuricemia is closely related to the established conventional cardiovascular risk factors, such as DM, hypertension, older age, and male gender. Increasing evidence shows that UA may cause kidney disease, hypertension, high oxidative stress, and metabolic syndrome [23,24,25]. Second, UA can lead to endothelial dysfunction and induce hypertension through activation of the renin-angiotensin system [26,27]. UA can cause rheological disturbances, such as increased platelet aggregation and adhesiveness [28]. Third, hyperuricemia has been reported to accelerate residual renal function decline which is a strong predictor of patient mortality in PD patients [29].

On the other hand, four possible explanations may explain why lower UA levels are associated with higher mortality rates. First, serum UA level may reflect the nutritional status because the major source of UA is derived from dietary intake of purines and nucleotides. Therefore, a low serum UA may indicate malnourished status, which in turn accounts for susceptibility to sepsis and impaired immune function [30]. In this study, we were able to show that low UA was associated with potential biomarkers of protein-energy wasting, such as low levels of serum albumin and phosphate. The presence of malnutrition and protein wasting is often accompanied by increased inflammation and oxidative stress, which has been linked with a high risk of mortality in ESRD patients [31,32]. Second, in both in vivo and in vitro experiments, UA has been shown to be a powerful free radical scavenger which could benefit the CV systems [33,34]. Third, the so-called “reverse epidemiology”, also known as “risk factor reversal”, cannot be completely excluded. Factors that are traditionally considered to be harmful indicators in the general population may be associated with beneficial effects on the clinical outcomes of various chronic diseases, such as obesity paradox in ESRD. When many possible confounding risk factors have been adjusted in the analysis, the paradoxical association of low UA level with high mortality remains significant in our study. Fourth, we lacked the data on C-reactive protein due to the retrospective nature of this study. It is unknown whether the results remain consistent if C-reactive protein is incorporated into the analysis. Fifth, inflammatory cytokines appear to cause low UA as a result of impaired net renal tubular reabsorption of urate [35]. A Taiwanese study of patients with severe acute respiratory syndrome showed that marked renal hypouricemia due to a defect in renal UA handling was associated with a higher serum IL-8 level [36]. However, this hypothesis may not affect ESRD patients to a great extent due to their small residual renal function, and we did not measure renal fractional excretion of UA in this study.

The strengths of our study include a medium-size population of CAPD patients. Since detailed information was collected regarding patient demographic features, comorbidities, medications, and laboratory data, we were able to conduct a comprehensive analysis to minimize the potential bias in observational studies. Some potential limitations of the present study should nonetheless be mentioned. First, all the patients were recruited from a single PD center and thus it does not allow the generalization of the results to the entire population of CAPD patients due to the diverse differences in cultural and demographic factors. Second, residual confounding factors cannot be completely excluded. For example, insulin resistance, which has been shown to be a predictor of cardiovascular mortality in ESRD patients, was not evaluated in our study. Third, due to the retrospective nature of this study, we were unable to determine whether the low UA level contributes to the reduced antioxidant capacity. Whether UA per se is a causative factor or merely a marker of nutrition, inflammation or oxidative stress cannot be answered from this study. Fourth, a single measurement of UA at baseline, which did not cover acute change in UA during the study period, might underestimate or over-estimate the true relationship with study outcomes. Despite these limitations, we believe this study contributes to the rather limited knowledge of the predictive value of UA in CAPD patients.

In conclusion, we found an inverse relationship between UA level and all-cause, CV, and infection-associated mortality in female patients on CAPD. Based on our findings, the beneficial impact associated with UA appears to outweigh its harmful effects, and may thus have a protective role in CAPD patients. This significant association should alert clinicians to pay more attention to women on CAPD with low UA levels. This UA paradox may be explained by the association between a low level of UA and poor nutrition and possibly increased oxidative stress. However, whether a low level of UA is the cause or consequence of these conditions remains to be elucidated. Further prospective studies of larger sample size are needed to confirm this relationship and to clarify the underlying mechanisms such as the role of UA in antioxidant capacity.

## Figures and Tables

**Table 1:** Baseline characteristics of the study population by sex-specific uric acid tertiles (*n* = 492).

	Tertile 1 (*n* = 170)	Tertile 2 (*n* = 163)	Tertile 3 (*n* = 159)	*p*-Value
Sex, men	80 (47.1%)	82 (50.3%)	74 (46.5%)	0.76
Age (years)	58 ± 14.8	52.9 ± 14	49.4 ± 15.8	<0.001
Body mass index (kg/m^2^)	22.7 (20.6–25.2)	23.8 (21.1–26.7)	23.2 (20.7–26.6)	0.09
**Smoker**				0.45
never	134 (78.8%)	137 (84.1%)	131 (82.4%)	
current	5 (2.9%)	5 (3.1%)	2 (1.3%)	
ever	31 (18.2%)	21 (12.9%)	26 (16.4%)	
**Status before PD**				0.17
pre-dialysis	132 (77.7%)	142 (87.1%)	129 (81.1%)	
hemodialysis	38 (22.4%)	19 (11.7%)	30 (18.9%)	
Failed transplant	0 (0%)	2 (1.2%)	0 (0%)	
**Medications use**				
ACE inhibitor/ARB	106 (62.4%)	110 (67.5%)	97 (61%)	0.44
Allopurinol	42 (24.7%)	46 (28.2%)	38 (23.9%)	0.64
Furosemide	78 (45.9%)	75 (46%)	78 (49.1%)	0.81
statin	34 (20%)	34 (20.9%)	35 (22%)	0.91
Erythropoiesis stimulating agents	155 (91.2%)	157 (96.3%)	153 (96.2%)	0.06
vitamin D	159 (93.5%)	153 (93.9%)	148 (93.1%)	0.96
Calcium channel blocker	117 (68.8%)	116 (71.2%)	113 (71.1%)	0.87
**Laboratory data**				
Uric acid (mg/dL)	6 (5.2–6.4)	7.3 (6.9–7.5)	8.7 (8.2–9.6)	<0.001
Serum albumin (g/dL)	3.3 (2.9–3.7)	3.4 (3–3.9)	3.4 (3.1–3.9)	0.01
Hemoglobin (g/dL)	8.79 (8–9.5)	8.7 (7.8–9.4)	8.6 (7.4–9.4)	0.09
BUN (mg/dL)	76.5 (64–90)	82 (68–96)	92 (76–109)	<0.001
Creatinine (mg/dL)	8.8 (7.6–11)	9.7 (8.1–11.9)	10 (8.5–12.8)	<0.001
Alkaline phosphatase (U/L)	101 (73–150)	98 (68–159)	97 (64–161)	0.68
Calcium (mg/dL)	8.3 (7.9–8.9)	8.4 (8–8.9)	8.3 (7.8–8.6)	0.18
Phosphorus (mg/dL)	5 (4.2–5.7)	5.3 (4.6–6.1)	5.9 (5.2–6.7)	<0.001
Cholesterol (mg/dL)	176 (152–209)	185 (154–219)	185 (151–217)	0.41
Triglyceride (mg/dL)	110.5 (83–151)	122 (96–173)	127 (91–182)	0.005
WBC count (×10^3^/μL)	7.1 (5.7–8.5)	7.2 (5.9–8.8)	6.9 (5.6–8.9)	0.75
Ferritin (ng/mL)	302.7 (158.5–550)	260.2 (127.6–421.7)	259 (136–550.6)	0.18
Transferrin saturation (%)	26.5 (17.62–35.39)	22.61 (16.72–31.31)	24.24 (17.12–34.59)	0.08
GPT (U/L)	16 (12–24)	15 (12–22)	16 (12–23)	0.92
Intact PTH (pg/mL)	312.17 (184–445.5)	350.8 (200.67–544)	375.8 (196–641)	0.06
**Comorbidities**				
Hypertension	146 (85.9%)	139 (85.3%)	134 (84.3%)	0.92
Diabetes mellitus	66 (38.8%)	64 (39.3%)	49 (30.8%)	0.21
Cardiovascular disease	69 (40.6%)	64 (39.3%)	53 (33.3%)	0.36
Charlson index	3 (2–4)	3 (2–4)	2 (2–4)	0.12
**Peritoneal dialysis-related data**				
D/P Creatinine at 4 h	0.7 (0.62–0.76)	0.67 (0.6–0.77)	0.65 (0.59–0.73)	0.01
Ultrafiltration (L/day)	0.22 (−0.2–0.6)	0.35 (−0.1–0.75)	0.5 (0.1–0.98)	<0.001
24 h urine volume (L)	0.93 (0.53–1.35)	0.88 (0.5–1.3)	0.85 (0.46–1.22)	0.35
Total Weekly Kt/V	2.14 (1.79–2.37)	2.03 (1.75–2.32)	2.01 (1.77–2.27)	0.31
nPNA (g/kg/day)	1.01 (0.82–1.2)	1.02 (0.8–1.22)	1.05 (0.89–1.26)	0.13
Residual renal function (mL/min/1.73 m^2^)	2.96 (2.13–3.68)	2.96 (2.13–3.82)	2.94 (1.96–3.74)	0.82

Values are expressed as mean ± standard deviation, median (interquartile range) or number (percentage). Abbreviations: PD, peritoneal dialysis; ACE inhibitor, angiotensin-converting enzyme inhibitor; ARB, angiotensin II receptor blocker; GPT, glutamic-pyruvic transaminase; WBC count, white blood cell count; PTH, parathyroid hormone; BUN, blood urea nitrogen; D/P creatinine, dialysate-to-plasma creatinine ratio; nPNA, normalized protein nitrogen appearance.

**Table 2:** Cox regression models of all-cause, cardiovascular and infection associated mortality for uric acid (sex-specific tertiles).

	Tertiles of Uric Acid	All-Cause Mortality		Cardiovascular Mortality		Infection-Related Mortality	
		HR (95% CI)	*p*-Value	HR (95% CI)	*p*-Value	HR (95% CI)	*p*-Value
Unadjusted	Tertile 2	0.71 (0.48–1.04)	0.08	0.74 (0.45–1.23)	0.25	0.48 (0.23–0.98)	0.04
	Tertile 3	0.32 (0.19–0.52)	<0.0001	0.33 (0.17–0.63)	0.001	0.31 (0.13–0.72)	0.007
Model 1	Tertile 2	1.03 (0.69–1.54)	0.87	0.99 (0.59–1.67)	0.97	0.81 (0.37–1.66)	0.58
	Tertile 3	0.49 (0.29–0.82)	0.006	0.44 (0.23–0.86)	0.02	0.52 (0.21–1.16)	0.14
Model 2	Tertile 2	1.02 (0.68–1.51)	0.94	1.03 (0.61–1.72)	0.92	0.83 (0.38–1.71)	0.62
	Tertile 3	0.46 (0.27–0.76)	0.003	0.48 (0.25–0.93)	0.03	0.57 (0.23–1.29)	0.21
Model 3	Tertile 2	0.88 (0.59–1.32)	0.53	0.91 (0.54–1.53)	0.72	0.81 (0.37–1.69)	0.59
	Tertile 3	0.4 (0.24–0.67)	0.001	0.39 (0.2–0.77)	0.006	0.53 (0.21–1.19)	0.15
Model 4	Tertile 2	0.83 (0.55–1.24)	0.36	1.01 (0.6–1.69)	0.97	0.83 (0.38–1.73)	0.63
	Tertile 3	0.35 (0.21–0.59)	<0.0001	0.38 (0.19–0.74)	0.005	0.43 (0.17–0.97)	0.06
Model 5	Tertile 2	1.06 (0.7–1.58)	0.8	1.04 (0.62–1.77)	0.87	0.9 (0.41–1.94)	0.8
	Tertile 3	0.4 (0.24–0.68)	0.001	0.4 (0.2–0.81)	0.01	0.47 (0.19–1.09)	0.1

Note: reference group is the tertile 1 of uric acid; HR: hazard ratio; CI: confidence interval; Model 1: adjusted for age, sex, body mass index, the pre-dialysis status, and smoking status; Model 2: adjusted for model 1 covariates and medications (angiotensin-converting enzyme (ACE) inhibitors/angiotensin II receptor blocker (ARB), erythropoiesis stimulating agents, furosemide, vitamin D, statin, allopurinol and calcium channel blocker); Model 3: adjusted for model 2 covariates and comorbidities (diabetes mellitus, hypertension, cardiovascular disease, and Charlson score); Model 4: adjusted for model 3 covariates and peritoneal dialysis (PD) related parameters (weekly total Kt/V urea, normalized protein nitrogen appearance (nPNA), dialysate-to-plasma (D/P) creatinine at 4 h, ultrafiltration, 24-h urine output, and residual renal function.); Model 5: adjusted for model 4 covariates and laboratory data (blood urea nitrogen (BUN), creatinine, albumin, glutamic-pyruvic transaminase (GPT), white blood cell (WBC) counts, alkaline phosphate, hemoglobin, ferritin, transferrin saturation, cholesterol, triglyceride, intact parathyroid hormone (PTH), calcium, and phosphate).

**Table 3:** Cox regression models of all-cause, cardiovascular, and infection associated mortality for uric acid (non-sex-specific tertiles).

	Tertiles of Uric Acid	All-Cause Mortality		Cardiovascular Mortality		Infection-Related-Mortality	
		HR (95% CI)	*p*-Value	HR (95% CI)	*p*-Value	HR (95% CI)	*p*-Value
Unadjusted	Tertile 2	0.74 (0.50–1.11)	0.15	0.80 (0.47–1.35)	0.41	0.53 (0.26–1.08)	0.08
	Tertile 3	0.35 (0.21–0.56)	<0.001	0.39 (0.21–0.72)	0.003	0.21 (0.08–0.55)	0.002
Model 1	Tertile 2	0.97 (0.64–1.45)	0.87	0.99 (0.58–1.69)	0.96	0.88 (0.42–1.83)	0.73
	Tertile 3	0.52 (0.32–0.85)	0.01	0.51 (0.27–0.97)	0.04	0.40 (0.15–1.05)	0.06
Model 2	Tertile 2	0.95 (0.64–1.43)	0.81	0.86 (0.49–1.50)	0.60	0.90 (0.43–1.88)	0.78
	Tertile 3	0.51 (0.31–0.84)	0.007	0.46 (0.24–0.88)	0.02	0.46 (0.17–1.24)	0.12
Model 3	Tertile 2	0.82 (0.54–1.24)	0.34	0.89 (0.52–1.52)	0.67	0.79 (0.37–1.65)	0.53
	Tertile 3	0.43 (0.26–0.69)	0.001	0.42 (0.22–0.78)	0.007	0.39 (0.15–1.05)	0.06
Model 4	Tertile 2	0.86 (0.58–1.29)	0.47	0.98 (0.58–1.68)	0.96	0.59 (0.28–1.26)	0.17
	Tertile 3	0.36 (0.22–0.60)	<0.001	0.396 (0.21–0.75)	0.004	0.29 (0.11–0.78)	0.01
Model 5	Tertile 2	0.79 (0.52–1.21)	0.28	0.98 (0.57–1.69)	0.96	0.58 (0.27–1.23)	0.15
	Tertile 3	0.43 (0.26–0.72)	0.001	0.45 (0.24–0.86)	0.01	0.24 (0.09–0.69)	0.008

Note: reference group is the tertile 1 of uric acid; HR: hazard ratio; CI: confidence interval; Model 1: adjusted for age, sex, body mass index, the pre-dialysis status, and smoking status; Model 2: adjusted for model 1 covariates and medications (angiotensin-converting enzyme (ACE) inhibitors/angiotensin II receptor blocker (ARB), erythropoiesis stimulating agents, furosemide, vitamin D, statin, allopurinol, and calcium channel blocker); Model 3: adjusted for model 2 covariates and comorbidities (diabetes mellitus, hypertension, cardiovascular disease, and Charlson score); Model 4: adjusted for model 3 covariates and peritoneal dialysis (PD) related parameters (weekly total Kt/V urea, normalized protein nitrogen appearance (nPNA), dialysate-to-plasma (D/P) creatinine at 4 h, ultrafiltration, 24-h urine output, and residual renal function.); Model 5: adjusted for model 4 covariates and laboratory data (blood urea nitrogen (BUN), creatinine, albumin, glutamic-pyruvic transaminase (GPT), white blood cell (WBC) counts, alkaline phosphate, hemoglobin, ferritin, transferrin saturation, cholesterol, triglyceride, intact parathyroid hormone (PTH), calcium, and phosphate).

**Table 4:** Sensitivity analyses of mortality risk for uric acid ^a^.

	All-Cause Mortality			Cardiovascular Mortality			Infection-Associated Mortality		
	HR (95% CI)	*p*-Value	*p* for Interaction	HR (95% CI)	*p*-Value	*p* for Interaction	HR (95% CI)	*p*-Value	*p* for Interaction
	mortality risk for every 1-mg/dL increase in serum uric acid level								
	Total cohort								
All patients (*n* = 492)	0.79 (0.69–0.9)	0.001	0.25	0.8 (0.68–0.96)	0.01	0.44	0.67 (0.53–0.84)	0.001	0.37
Men	0.84 (0.69–1.02)	0.07		0.79 (0.61–1.01)	0.06		0.79 (0.48–1.22)	0.32	
Women	0.57 (0.44–0.73)	<0.0001		0.6 (0.41–0.87)	0.006		0.41 (0.26–0.59)	<0.0001	
	Excluding those taking allopurinol								
All patients (*n* = 366)	0.78 (0.65–0.94)	0.009	0.79	0.7 (0.52–0.93)	0.01	0.94	0.66 (0.5–0.85)	0.002	0.49
Men	0.7 (0.44–1.1)	0.12		0.63 (0.4–1.01)	0.05		0.63 (0.38–1.04)	0.07	
Women	0.47 (0.31–0.7)	<0.0001		0.54 (0.34–0.79)	0.004		0.41 (0.24–0.63)	<0.0001	
	Hyperuricemia vs. non-hyperuricemia								
All patients	0.55 (0.37–0.82)	0.003	0.18	0.52 (0.31–0.87)	0.01	0.52	0.46 (0.26–0.59)	0.02	0.09
Men (>7 mg/dL)	0.63 (0.37–1.05)	0.08		0.53 (0.26–1.08)	0.08		0.64 (0.38–1.08)	0.1	
Women (>6 mg/dL)	0.29 (0.15–0.58)	0.001		0.28 (0.11–0.7)	0.007		0.48 (0.26–0.92)	0.03	

HR: hazard ratio; CI: confidence interval; ^a^ Adjusted for model 5 covariates in Table 2.

**Table 5:** Multivariate linear regression of serum uric acid levels with clinical parameters at baseline.

	Unstandardized Coefficients		Standardized Coefficients Beta	*p*-Value
	B	Std. Error		
Sex (male vs. female)	0.280	0.137	0.088	0.04
Age	−0.013	0.005	−0.121	0.01
Albumin	0.463	0.132	0.159	0.001
Alkaline phosphate	−0.002	0.001	−0.084	0.05
BUN	0.008	0.003	0.119	0.02
Intact PTH	0.000	0.000	0.097	0.04
Phosphorus	0.225	0.066	0.186	0.001
Triglyceride	0.003	0.001	0.183	<0.001
WBC counts	0.053	0.028	0.080	0.06
Cardiovascular disease	0.347	0.146	0.106	0.02
Ultrafiltration	0.155	0.090	0.073	0.09
24-h urine output	−0.575	0.166	−0.216	0.001
Residual renal function	0.21	0.058	0.216	<0.001

Abbreviations: WBC, white blood cell count; PTH, parathyroid hormone; BUN, blood urea nitrogen.

**Table 6:** Stratified analyses by various nutritional markers.

	UA Tertiles	All-Cause Mortality			Cardiovascular Mortality			Infection-Mortality		
		aHR (95% CI)	*p*-Value	*P*_interaction_	aHR (95% CI)	*p*-Value	*P*_interaction_	aHR (95% CI)	*p*-Value	*P*_interaction_
BMI ≥ 23.1	Tertile 2	0.93 (0.52–1.68)	0.82	0.39	0.94 (0.49–1.81)	0.85	0.34	1.11 (0.16–7.9)	0.91	0.43
	Tertile 3	0.38 (0.17–0.86)	0.02	0.41	0.24 (0.09–0.66)	0.006	0.22	0.51 (0.07–3.54)	0.49	0.38
BMI < 23.1	Tertile 2	1.16 (0.64–2.09)	0.63		1.17 (0.48–2.85)	0.72		1.12 (0.38–3.33)	0.83	
	Tertile 3	0.33 (0.15–0.71)	0.005		0.39 (0.13–1.11)	0.08		0.86 (0.23–3.21)	0.83	
Albumin ≥ 3.4	Tertile 2	1.32 (0.65–2.69)	0.44	0.81	1.08 (0.42–2.75)	0.87	0.81	2.02 (0.46–8.78)	0.34	0.10
	Tertile 3	0.38 (0.15–0.92)	0.03	0.14	0.48 (0.16–1.43)	0.18	0.64	0.81 (0.10–6.38)	0.84	0.36
Albumin < 3.4	Tertile 2	0.80 (0.49–1.33)	0.39		0.94 (0.48–1.81)	0.85		0.48 (0.17–1.37)	0.17	
	Tertile 3	0.47 (0.25–0.90)	0.02		0.54 (0.23–1.27)	0.15		0.39 (0.13–1.19)	0.09	
Cholesterol ≥ 181	Tertile 2	1.17 (0.64–2.16)	0.61	0.43	1.09 (0.52–2.32)	0.81	0.48	1.05 (0.17–6.38)	0.96	0.28
	Tertile 3	0.59 (0.26–1.34)	0.21	0.85	0.47 (0.16–1.35)	0.16	0.41	0.87 (0.16–4.76)	0.87	0.75
Cholesterol < 181	Tertile 2	0.68 (0.36–1.28)	0.22		0.55 (0.22–1.37)	0.20		0.88 (0.35–2.22)	0.79	
	Tertile 3	0.41 (0.20–0.84)	0.01		0.54 (0.21–1.36)	0.19		0.51 (0.16–1.56)	0.24	
nPNA ≥ 1.03	Tertile 2	1.02 (0.47–2.24)	0.95	0.09	0.42 (0.15–1.16)	0.09	0.47	0.86 (0.16–4.64)	0.86	0.28
	Tertile 3	0.39 (0.15–1.01)	0.05	0.96	0.25 (0.07–0.88)	0.03	0.95	0.93 (0.21–4.17)	0.93	0.59
nPNA < 1.03	Tertile 2	1.16 (0.71–1.92)	0.55		0.87 (0.35–2.19)	0.77		0.78 (0.3–2.03)	0.61	
	Tertile 3	0.56 (0.29–1.08)	0.08		0.43 (0.13–1.44)	0.17		0.43 (0.14–1.32)	0.14	
Phosphorus ≥ 5.4	Tertile 2	0.96 (0.41–2.24)	0.92	0.21	1.03 (0.28–3.78)	0.96	0.99	1.05 (0.19–5.71)	0.95	0.04
	Tertile 3	0.26 (0.09–0.69)	0.007	0.23	0.50 (0.14–1.76)	0.28	0.63	0.59 (0.08–4.32)	0.60	0.51
Phosphorus < 5.4	Tertile 2	0.84 (0.51–1.36)	0.47		0.86 (0.44–1.67)	0.65		0.45 (0.17–1.17)	0.10	
	Tertile 3	0.54 (0.28–1.01)	0.05		0.36 (0.14–0.94)	0.03		0.41 (0.14–1.15)	0.09	
Creatinine ≥ 9.6	Tertile 2	0.89 (0.44–1.83)	0.76	0.39	0.87 (0.28–2.77)	0.82	0.97	0.51 (0.09–2.58)	0.41	0.11
	Tertile 3	0.41 (0.15–1.09)	0.07	0.99	0.21 (0.04–1.16)	0.07	0.96	0.51 (0.08–3.18)	0.47	0.56
Creatinine < 9.6	Tertile 2	0.82 (0.49–1.36)	0.45		0.96 (0.46–2.01)	0.91		0.51 (0.21–1.28)	0.15	
	Tertile 3	0.39 (0.21–0.75)	0.004		0.39 (0.15–1.04)	0.06		0.56 (0.17–1.81)	0.33	

Note: reference group is the tertile 1 of uric acid. The median values of these parameters were used to divide the entire cohort into two subgroups. Abbreviations: UA, uric acid; BMI, body mass index; nPNA, normalized protein nitrogen appearance; CI, confidence interval; aHR, adjusted hazard ratio.
